# Apoptosis-Inducing Effects of *Lactobacillus plantarum* DS0709 in Colorectal Cancer

**DOI:** 10.4014/jmb.2504.04042

**Published:** 2025-08-15

**Authors:** In Hwan Tae, Yunsang Kang, Jinkwon Lee, Jeongmin Lee, Jinsan Kim, Haneol Yang, Kunhyang Park, Doo-Sang Park, Dae-Soo Kim, Hyun-Soo Cho

**Affiliations:** 1 Korea Research Institute of Bioscience and Biotechnology, Daejeon 34141, Republic of Korea; 2 Korea University of Science and Technology, Daejeon 34113, Republic of Korea; 3 Department of Biological Science, Sungkyunkwan University, Suwon 16419, Republic of Korea

**Keywords:** *L. plantarum* DS0709, apoptosis, colorectal cancer

## Abstract

In colorectal cancer (CRC) treatment, various approaches, including chemotherapy (5-FU, irinotecan, and oxaliplatin), targeted therapy (VEGF inhibitor) and immunotherapy (PD-1/ PD-L1 inhibitor), are employed. However, due to side effects and limited efficacy, more effective novel therapeutic strategies have been required. In this study, we identified the anti-cancer effects of *Lactobacillus plantarum* DS0709, isolated from infant feces, on CRC. Treatment with the supernatant (Sup) of *L. plantarum* DS0709 demonstrated growth inhibition of CRC cell lines (HCT116 and SNUC5) by inducing apoptosis. Additionally, using human iPSC-derived intestinal organoids (hIO), we confirmed that *L. plantarum* DS0709 Sup exhibited no toxicity. Furthermore, in a 3D spheroid model mimicking *in vivo* conditions, *L. plantarum* DS0709 Sup showed similar apoptosis induction and growth-inhibitory effects as in 2D cultures. Thus, these findings suggest that *L. plantarum* DS0709 has the potential to be developed into a novel microbiome-based therapeutic agent for CRC, offering anti-cancer efficacy without side effects.

## Introduction

For the treatment of colorectal cancer (CRC), chemotherapy using 5-fluorouracil (5-FU) and irinotecan is commonly used [1]. However, due to severe side effects and limited efficacy, recent efforts have shifted toward targeted therapy and immunotherapy [2]. Unfortunately, these approaches also face challenges of low efficacy and side effects. Therefore, there is a growing demand for the development of novel therapeutic strategies to improve the efficacy of CRC treatment.

The gut microbiome has been shown to be associated with various diseases [3-5]. Particularly, studies comparing the gut microbiome of CRC patients and healthy individuals have increasingly reported a significant correlation between gut microbiome and CRC development [6, 7]. Analysis of the gut microbiome has identified specific microbiota associated with the suppression and progression of CRC [8-11]. These findings suggest that various metabolites produced by the gut microbiota may play an essential role in suppressing CRC [12-14]. Recent studies have reported that short-chain fatty acids produced by the gut microbiota regulate the protein stability of histone methyltransferase euchromatic histone lysine n-methyltransferase 2 (EHMT2), thereby inhibiting CRC growth [15]. These findings highlight the potential of the gut microbiome as a therapeutic agent for CRC treatment. However, there remains a lack of research on identifying CRC-related microbiota and elucidating their mechanisms of action (MOA) in inhibition of CRC proliferation.

*Lactobacillus plantarum* is a Gram-positive bacterium that metabolizes sugars like lactose to produce lactic acid [16, 17]. The lactic acid produced by *L. plantarum* in human gut decrease the surrounding pH and inhibits the growth of harmful bacteria [18]. Recent studies have reported that *L. plantarum* suppresses CRC growth and enhances chemosensitivity when administered in combination with chemotherapy agents, such as 5-FU [19].

In this study, we identified *L. plantarum* DS0709 from infant feces. To investigate its anti-cancer effects, we used HCT116 and SNUC5 CRC cell lines and demonstrated that treatment with *L. plantarum* DS0709 supernatant (sup) induces apoptosis, thereby inhibiting cell proliferation. Additionally, we conducted toxicity tests to confirm the safety of *L. plantarum* DS0709 using human induced pluripotent stem cell (hiPSC)-derived intestinal organoids. Based on these findings, *L. plantarum* DS0709 exhibits no side effects and effectively suppresses CRC growth, suggesting its potential application as a gut microbiome-based therapeutic agent for CRC treatment. Furthermore, its combined use with various chemotherapy agents could lead to enhanced therapeutic efficacy in CRC treatment.

## Materials and Methods

### Cell Culture

The colorectal cancer cell lines were obtained from either the American Type Culture Collection (ATCC, USA), HCT116, or the Korean Cell Line Bank (KCLB, Republic of Korea), SNUC5. The cells were maintained in RPMI-1640 medium (Cat. no. LM011-01, Welgene, Republic of Korea) supplemented with 10% fetal bovine serum (FBS; Cat. no. 10082147, Gibco, USA) and 1% penicillin/streptomycin (Cat. no. 15140122, Gibco). All cells were cultured in a humidified atmosphere containing 5% CO_2_ at 37°C.

### Bacterial Culture

The *Lactobacillus plantarum* strain was obtained from the Korean Collection for Type Cultures (KCTC13636BP). The bacterial strain was cultivated in de Man, Rogosa and Sharpe (MRS) media (Cat. no. 288210, BD, USA) under anaerobic condition at 37°C for 36 h. The bacterial culture was incubated at 65°C for 30 min for Pasteurization and centrifuged at 3,000 g for 10 min. The supernatant was collected in a fresh new tube and kept at -70°C until use.

### 3D Spheroid Culture

Spheroid cultures of colorectal cancer cell lines were established using ultra-low attachment plates (ULA plate; Cat. no. 7007, Corning, USA). For each well, 5 × 10^4^ HCT116 and SNUC5 cells were seeded and maintained for 24 h. The spheroids were then exposed to *L. plantarum* DS0709 supernatant and cultured for an additional 72 h [20]. Spheroid formation was monitored every 24 h using an Olympus microscope (Cat. no. CKX53, Olympus, Japan).

### Human Intestinal Organoid Culture

The human intestinal organoids (KCTC 3D 0011, passage 2) used in this study were obtained from the Korean Collection for Type Cultures (KCTC), supported by the Ministry of Food and Drug Safety under the project " Development of Organoid-Based Animal Alternative Resource Bank Establishment and Operation System" (RS-2024-00332162). The organoids were cultured in advanced DMEM/F12 medium (Cat. no. 12634010, Gibco) supplemented with 100 ng/ml epidermal growth factor (Cat. no. 236-EG, R&D Systems, USA), 500 ng/ml R-spondin1 (Cat. no. 4645-RS, R&D Systems), 100 ng/ml Noggin (Cat. no. 3344-NG, R&D Systems), and 1X B27 supplement (Cat. no. 17504044, Gibco). The medium was refreshed every two days.

### Cell Viability Assay

Cells were seeded in 6-well plates at a density of 1 × 10^5^ cells per well (HCT116) or 2.5 × 10^5^ cells per well (SNUC5) and incubated overnight. After 72 h of *L. plantarum* DS0709 supernatant treatment, a mixture of Cell Counting Kit-8 (CCK-8; Cat. no. E-CK-A362, Elabscience, USA) solution and cell culture medium (1 ml/well) was added, followed by incubation at 37°C for 5 min. The absorbance was measured at 450 nm using a microplate reader. For crystal violet staining, cells were fixed with 100% methanol for 5 min and stained with 0.1% crystal violet solution (Cat. no. C0775, Sigma Aldrich, USA).

### Fluorescence-Activated Cell Sorting (FACS) Analysis

After treatment with DS0709 supernatant, cells were collected and stained with the Muse Annexin V and Dead Cell Assay kit (Cat. no. MCH100105, Merck, Germany) for 20min at room temperature. For caspase analysis, cells were incubated with the Muse Caspase 3/7 Kit (Cat. no. MCH100108, Merck): first with the Caspase 3/7 reagent for 30min at 37°C in a humidified 5% CO_2_ incubator, followed by staining with Caspase 7-AAD for 5min at room temperature. Approximately 5×10^4^ cells were then analyzed using a Muse Cell Analyzer (Cat. no. 0500-3115, Merck), and data were processed using Muse 1.6 Analysis Software.

### PI Staining

For detection of cell death, spheroids were incubated for 24 h after seeding, then stained with propidium iodide (PI; Cat. no. P3566, Invitrogen, USA) and treated with *L. plantarum* DS0709 supernatant. The stained spheroids were visualized using CELENA S Digital Cell Imaging System (Cat. no. CS20001, Logos Biosystems, Republic of Korea) to detect PI-positive dead cells. Both transmitted light and fluorescence images were captured to analyze spheroid morphology and cell death, respectively.

### RNA Sequencing Analysis

For total RNA-seq analysis, using TrueSeq RNA Sample Preparation Kit V2, purification and library construction were carried out with total RNA, and Illumina NextSeq 1000 machines (Illumina, 20038898) were used for sequencing, with a read length of 2 × 100 bases. A filtered read set was created using the Cutadapt v1.18 (https://cutadapt.readthedocs.io/en/stable/) command line parameters ‘-a AGATCGGAAGAGCACACGTCTGAACTC CAGTCAC -AAGATCGGAA GAGCGTCGTGTAGGGAAAGAGTGTA -m 50 -O 5’, and Sickle v1.33 (https://github.com/najoshi/sickle ) was used to remove the low-quality sequence (Phred score < 20) to a minimum length of 50 bp. We assessed the quality of the paired-end reads using FastQC version 0.11.4. Additionally, duplicate sequences were examined through the application of the FASTQC tool. The trimmed data containing low-quality reads and poly-N sequences were processed using the NGSQCToolkit v2.3.3 (https://github.com/mjain-lab/NGSQCToolkit). The reads were subsequently aligned to the human genome assembly GRCh38.97 (Accession No. GCA_000001405.27) by HISAT2 v2.1.0 (https://daehwankimlab.github.io/hisat2/). The obtained transcripts were quantified in fragments per kilobase million (FPKM) format using StringTie v2.2.1 (https://github.com/gpertea/stringtie) to calculate expression values and obtain normalized counts.

### Statistical Analysis

Statistical differences between the two groups were analyzed using an unpaired *t*-test. P-values obtained from the database were used to determine significance, indicated by *p < 0.05; **p < 0.01; ***p < 0.001.

## Results

### *L. plantarum* DS0709 Inhibits CRC Cell Growth

**Fig. 1:**
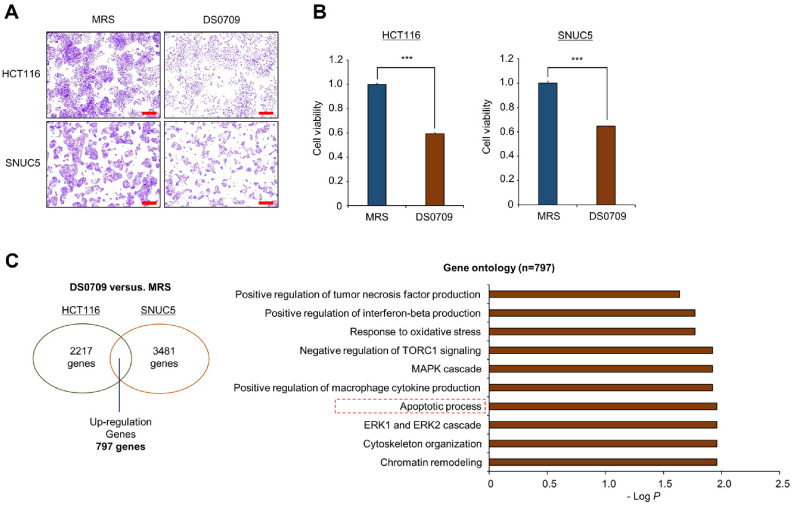
*L. plantarum* DS0709 sup suppressed cell growth in colon cancer cell lines. (**A**) Cell growth assay was conducted after 72 h of treatment with MRS and *L. plantarum* DS0709 sup. HCT116 and SNUC5 cells were fixed with ice-cold 100% methanol and stained using a 0.1% crystal violet solution. (scale bar, 200 μm). (**B**) CCK-8 assay. After adding the CCK-8 solution, the cells were incubated for 5 min at 37°C, and cell growth intensity was measured at 450 nm using a microplate reader. Data are presented as the mean ± SD from three independent experiments. All *p*-values were determined using Student's *t*-test (***p < 0.001). (**C**) GO analysis using DAVID (DAVID Knowledgebase v2024q4) was performed on RNA-seq data for 797 upregulated genes (fold change ≥ 1.2) shared between HCT116 and SNUC5 cell lines after 72 h treatment with MRS and *L. plantarum* DS0709 sup. Enriched GO terms are shown.

**Fig. 2:**
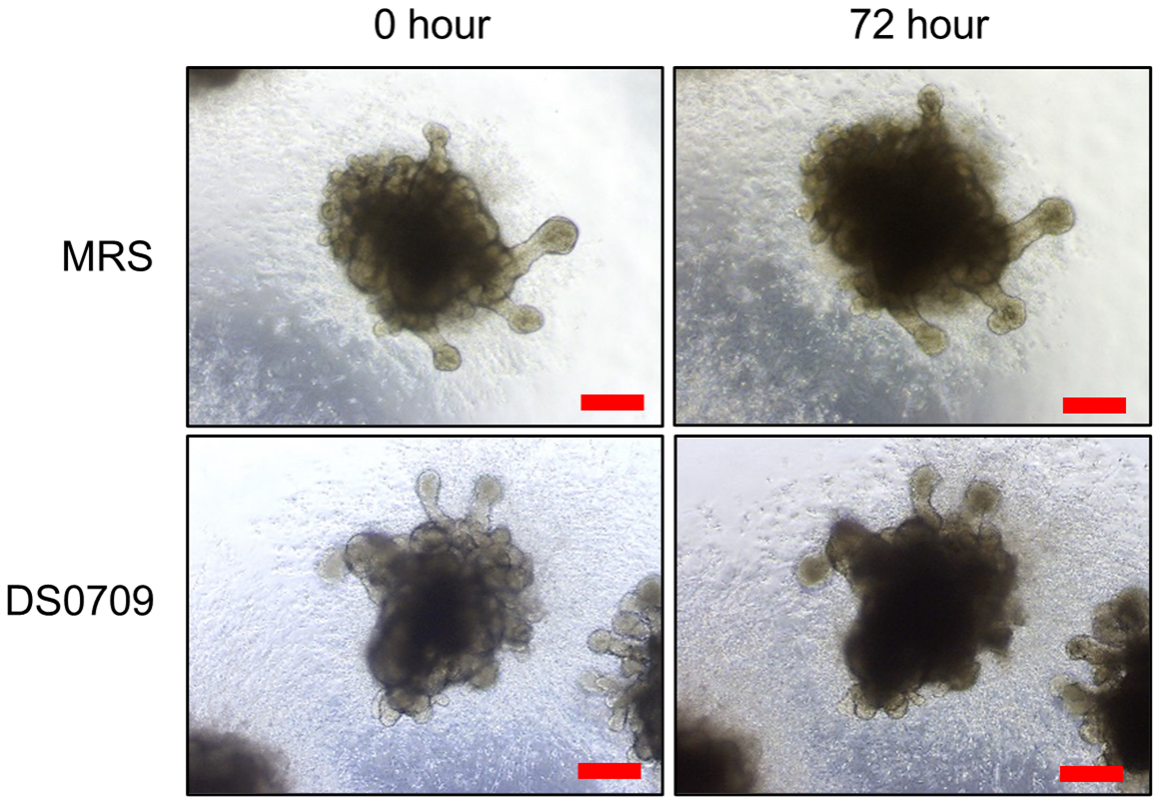
*L. plantarum* DS0709 sup exhibited no morphological effect in human intestinal organoids (hIOs). Representative images showing the morphology of hIOs following 72 h of treatment with MRS and *L. plantarum* sup. (scale bar, 200 μm).

To investigate the inhibitory effects of *L. plantarum* DS0709 on CRC cell growth, the supernatant of *L. plantarum* DS0709 was treated on HCT116 and SNUC5 cell lines. As shown in Fig. 1A, crystal violet (CV) staining revealed that *L. plantarum* DS0709 Sup significantly inhibited the growth of CRC cells compared to the negative control (MRS broth). Similarly, a Cell Counting Kit-8 (CCK-8) assay confirmed the growth-inhibitory effects of *L. plantarum* DS0709 Sup (Fig. 1B). Next, to understand the mechanism behind this growth inhibition, RNA sequencing (RNA-seq) was performed after treating the HCT116 and SNUC5 cells with *L. plantarum* DS0709 Sup. Gene Ontology (GO) term analysis with the RNA-seq results (*n* = 797) revealed that GO terms of “Apoptosis process”, “Positive regulation of tumor necrosis factor production” were clearly associated with *L. plantarum* DS0709 Sup, indicating that the treatment may be induced to cell apoptosis (Fig. 1C). To evaluate the effects of *L. plantarum* DS0709 Sup on normal cells, hiPSC-derived intestinal organoids (hIOs) were used. As shown in Figure 2, *L. plantarum* DS0709 Sup had no significant effect on the growth of hIOs, similar to the MRS control. These results demonstrate that *L. plantarum* DS0709 Sup inhibits the growth of CRC cells without affecting normal cells, suggesting its potential as a therapeutic agent for CRC. Furthermore, its combination with chemotherapy could enhance CRC treatment efficacy.

### *L. plantarum* DS0709 Sup Induces Cell Apoptosis in CRC Cells

**Fig. 3:**
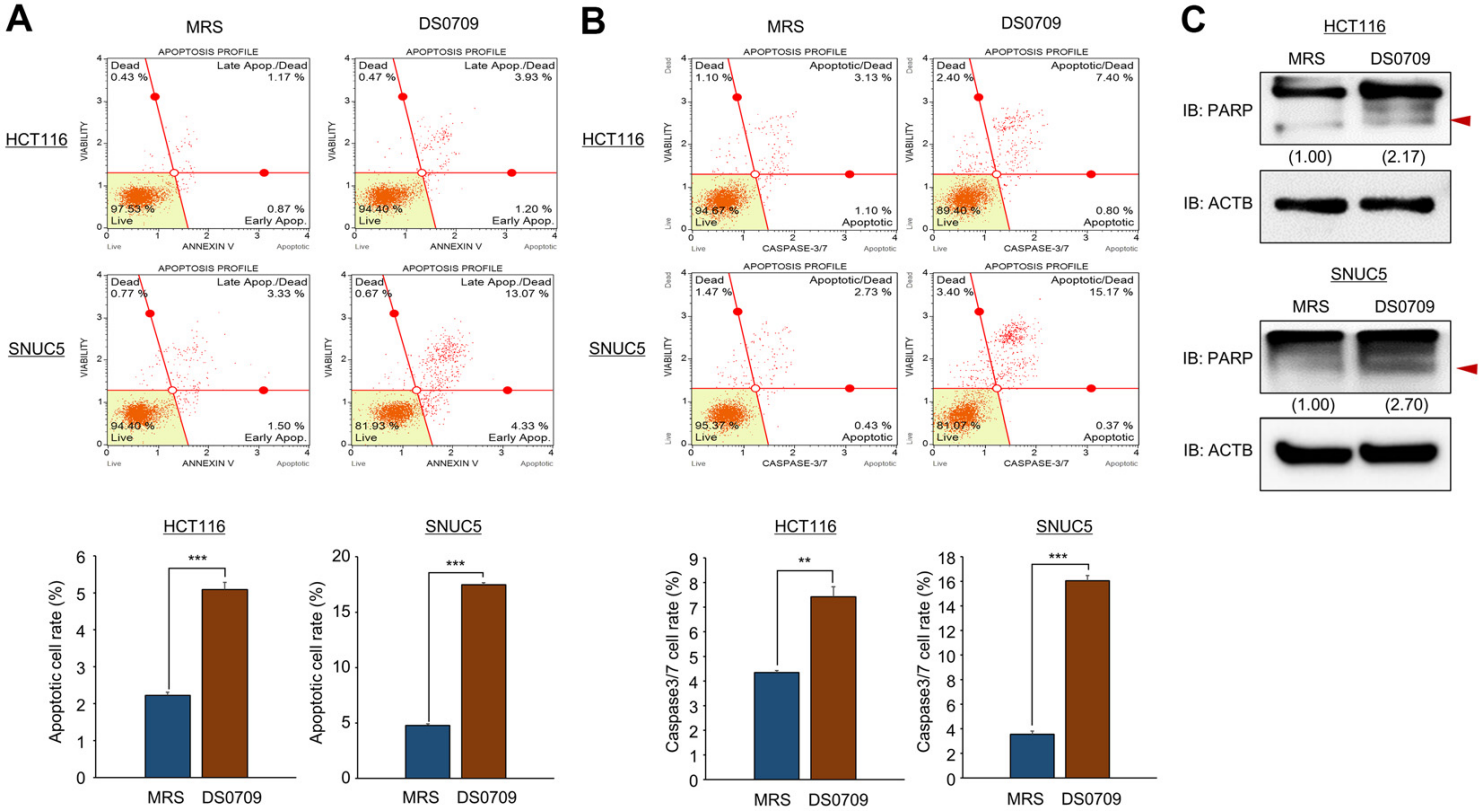
*L. plantarum* DS0709 sup triggered apoptotic cell death in colon cancer cell lines. (**A**) FACS analysis with PI staining was performed after 72 h of treatment with MRS and *L. plantarum* DS0709 sup. The lower right and upper right quadrants represent early and late apoptosis, respectively (upper). Quantification of apoptosis is displayed (lower). Data are presented as the mean ± SD from three independent experiments. All *p*-values were determined using Student's *t*-test (***p < 0.001). (**B**) FACS analysis with Muse Caspase 3/7 working solution was conducted after 72 h treatment with MRS and *L. plantarum* DS0709 sup. The upper right panel indicates the apoptotic and dead cell proportions (upper). Quantification of caspase 3/7 activity is shown (lower). Data are presented as the mean ± SD from three independent experiments. All *p*-values were determined using Student's *t*-test (***p < 0.001, **p<0.01). (**C**) Western blot analysis following *L. plantarum* DS0709 sup treatment using anti-PARP. ACTB was used as the internal control in CRC cell lines.

To confirm the apoptotic effects of *L. plantarum* DS0709 Sup on CRC cells, Annexin V FACS analysis was conducted. Treatment of HCT116 and SNUC5 cells with *L. plantarum* DS0709 Sup resulted in an increased proportion of apoptotic cells compared to the MRS control (Fig. 3A). Additionally, the activity of caspase-3/7, key mediators of the canonical apoptosis pathway, was significantly increased following treatment with *L. plantarum* DS0709 Sup compared to MRS control (Fig. 3B). Also, western blot analysis was used to measure status of cleaved PARP, a marker of apoptosis. As shown in Figure 3C, cleaved PARP status was elevated in cells treated with *L. plantarum* DS0709 Sup. Thus, these findings suggest that *L. plantarum* DS0709 Sup induces apoptosis, thereby inhibiting the growth of CRC cells.

### Growth Inhibition by *L. plantarum* DS0709 Sup in a 3D Spheroid Model

**Fig. 4:**
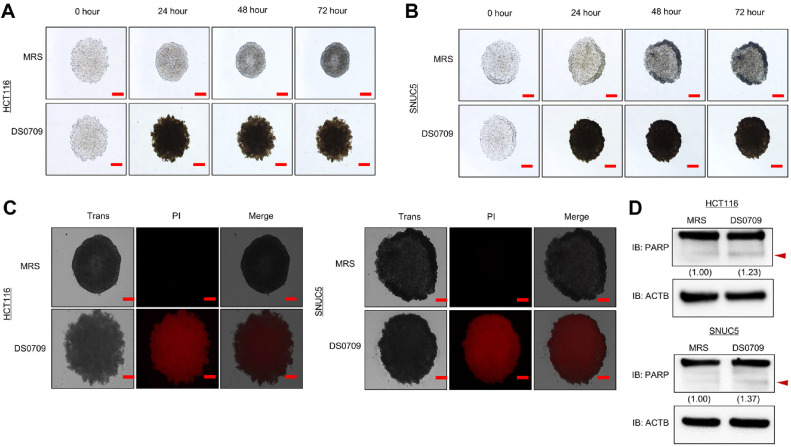
*L. plantarum* DS0709 sup promotes apoptosis in 3D spheroid colon cancer models. (**A** and **B**) 3D spheroid formation assay using the HCT116 and SNUC5 cell lines. Cells were seeded into ULA plates and treated with MRS and *L. plantarum* DS0709 sup for 72 h. The cells were imaged under a microscope every 24 h. (scale bar, 200 μm) (**C**) After 72 h of treatment with MRS and *L. plantarum* DS0709 sup, 3D spheroids were stained with propidium iodide (PI) and imaged using fluorescence microscopy (scale bar, 200 μm). (**D**) Western blot analysis following *L. plantarum* DS0709 sup treatment in 3D spheroids using anti-PARP. ACTB was used as the internal control in CRC cell lines.

The three-dimensional (3D) spheroid culture model is considered more representative of the *in vivo* environment compared to 2D culture [21]. To mimic *in vivo* conditions, HCT116 and SNUC5 cells were cultured using Ultra Low Attachment (ULA) plates. As shown in Fig. 4A and 4B, cells aggregated well to form spheroids in the ULA plates. The effects of *L. plantarum* DS0709 Sup on these 3D spheroids were then assessed. Treatment with *L. plantarum* DS0709 Sup caused dissociation of the spheroid aggregates (Fig. 4A and 4B). To evaluate apoptosis induction in 3D culture, propidium iodide (PI) staining was performed. After treating the 3D-cultured HCT116 and SNUC5 cells with *L. plantarum* DS0709 Sup, fluorescence measurements revealed an increased signal in treated samples compared to the MRS control (Fig. 4C). Next, western blot analysis further confirmed that *L. plantarum* DS0709 Sup increased the status of cleaved PARP in 3D cultures (Fig. 4D). These results demonstrate that *L. plantarum* *DS0709* Sup induces apoptosis and inhibits cell growth in the 3D spheroid model, similar to its effects in the 2D culture model. In conclusion, *L. plantarum* DS0709 Sup exhibits potent growth-inhibitory effects on CRC cells through apoptosis induction, even under *in vivo*-like conditions, highlighting its potential as a CRC therapeutic agent.

## Discussion

Metabolites produced by gut microbiota have been reported to be associated with the suppression of various diseases, including CRC [14]. Consequently, ongoing research is focused on screening metabolites and gut microbiota with cancer-specific inhibitory effects. Thus, in this study, we demonstrated that *L. plantarum* DS0709 induces apoptosis in CRC cell lines, leading to the inhibition of cancer cell growth. Furthermore, using hIO, we confirmed that *L. plantarum* DS0709 does not exhibit toxicity, suggesting its potential for selectively inhibiting CRC cell growth without affecting normal cells (Fig. 2).

However, further research is needed to identify the specific metabolites in *L. plantarum* DS0709 Sup responsible for inhibiting CRC cell growth. Metabolite analysis is essential for this purpose. Screening single metabolites can enable the more effective application of *L. plantarum* DS0709 and provide insights into potential side effects for CRC treatment. Additionally, further studies are required to investigate the mode of action (MOA) associated with the single metabolites in suppression of CRC growth. In addition, the efficacy of *L. plantarum* DS0709 could be further validated through *in vivo* studies using mouse models. *In vivo* validation would complement the mechanistic insights obtained from studies using individual *L. plantarum* DS0709 metabolites, particularly those metabolites that were shown to exert anti-tumor effects *in vitro*. Detailed investigation into the MOA of these single metabolites would provide a clearer understanding of how *L. plantarum* DS0709 exerts its beneficial effects at the molecular level. Moreover, the combination of *in vivo* mouse experiments and *in vitro* hIO models for toxicity and mechanistic studies creates a robust translational pipeline. This integrated approach enhances the reliability of preclinical data and supports the therapeutic potential of *L. plantarum* DS0709 as a candidate microbiome-based intervention for colorectal cancer.

Using RNA-seq data obtained after treatment with L.plantarum DS0709, we performed a GO term analysis and identified not only the "apoptotic process" but also a variety of other relevant biological terms. Notably, the term "cytoskeleton organization" was enriched, suggesting that *L. plantarum* DS0709 may influence the migratory capacity of CRC cell lines. This observation implies a potential role in suppressing metastasis, thereby indicating that *L. plantarum* DS0709 might contribute not only to tumor growth inhibition but also to the regulation of cancer cell dissemination. Furthermore, enrichment of the term "chromatin remodeling" implies a possible involvement of *L. plantarum* DS0709 in epigenetic regulation. Among such mechanisms, histone methyltransferases—key enzymes responsible for histone methylation—have emerged as critical factors in cancer progression and metastasis, not only in CRC but across multiple cancer types, making them important targets for therapeutic development [22-25]. Based on these findings, it is plausible that *L. plantarum* DS0709 may exert its anti-cancer effects, by modulating epigenetic regulators, including histone methyltransferases. These results collectively enhance the potential of *L. plantarum* DS0709 as a promising therapeutic candidate for colorectal cancer treatment.

In this study, we comprehensively assessed the safety profile of *L. plantarum* DS0709 by employing hIOs as an *in vitro* model system. While conventional safety testing often utilizes immortalized normal epithelial cell lines, these models are limited in their ability to represent the complex multicellular environment of the human intestine. In contrast, hIOs are derived from human pluripotent stem cells and can differentiate into various specialized intestinal cell types—including goblet cells, paneth cells, enteroendocrine cells, and enterocytes—offering a more physiologically relevant and dynamic model [26]. Although the hIOs used in this study were derived from small intestinal tissue and may not exactly mirror the cellular composition of colon, they still harbor key cell types found in both regions, such as goblet cells. This cellular diversity enables a more accurate evaluation of potential cytotoxicity, compared to monocultures of a single epithelial cell type. By applying *L. plantarum* DS0709 to these organoids and monitoring for morphological changes, we found no significant adverse effects, thereby confirming the safety of *L. plantarum* DS0709. These findings not only support the potential use of *L. plantarum* DS0709 as a candidate therapeutics for CRC, but also highlight the utility of hIOs as a preclinical platform for assessing the safety of next-generation microbiome-based therapeutics. Furthermore, this approach allows for the evaluation of microbial toxicity across a range of concentrations, ultimately contributing to the development of safer, dose-optimized microbial therapies for CRC and possibly other gastrointestinal diseases. Moreover various chemotherapy agents, such as 5-FU and irinotecan, are commonly used for CRC treatment [27]. The use of *L. plantarum* DS0709 in combination with these chemotherapeutic agents could potentially result in synergistic effects. Since *L. plantarum* DS0709 does not exhibit toxicity, its concurrent use with chemotherapy may allow for reduced dosages of chemotherapeutic agents while maintaining therapeutic efficacy. This could help mitigate the side effects associated with chemotherapy while enhancing treatment outcomes.

In conclusion, we suggested that *L. plantarum* DS0709 inhibits CRC cell growth by inducing apoptosis. These findings highlight its potential application in the development of microbial therapeutics for CRC treatment. Additionally, the combination of *L. plantarum* DS0709 with chemotherapeutic agents may provide a more effective approach to CRC therapy.
